# Denosumab and Sunitinib in the treatment of giant-cell tumor of bone with pulmonary and bone metastases in an adolescent: A case report

**DOI:** 10.1097/MD.0000000000017778

**Published:** 2019-11-15

**Authors:** Guannan Wang, Sujing Jiang, Zhouqi Li, Ying Dong

**Affiliations:** a Department of Medical Oncology, The Second Affiliated Hospital, College of Medicine, Zhejiang University, Hangzhou; b Department of Radiation and Medical Oncology, The First Affiliated Hospital, Wenzhou Medical University, Wenzhou, Zhejiang, China.

**Keywords:** Denosumab, giant cell tumor of bone, metastasis, spine, Sunitinib

## Abstract

**Introduction:**

Giant cell tumor of bone with pulmonary and bone metastases is exceedingly rare in adolescents. Furthermore, Denosumab and Sunitinib in the treatment of giant cell tumor of bone has never been reported.

**Patient concerns:**

A 16-year-old boy complained of fever, chest tightness, and shortness of breath and back pain for 5 days.

**Diagnosis:**

Giant cell tumor of bone with pulmonary and bone metastases.

**Interventions:**

The patient underwent 2 surgeries for giant cell tumor of bone located in the spine and received Denosumab to reduce local recurrence and control the metastases. Then Sunitinib was added into treatment strategies after the progressing of metastases.

**Outcomes:**

Within 5 months of Denosumab and Sunitinib, lung metastases were shrinking (stable disease, response evaluation criteria in solid tumors version 1.1). Until now about 4 years into treatment the patient is still survival. Pulmonary and bone metastases are stable.

**Conclusions:**

This is a case of multi-center giant cell tumor of bone, it does not only provide a reference to the treatment of similar cases of the clinic but also reflects the limitations of the application of Denosumab in the real world.

## 1 Introduction

Giant-cell tumor of bone (GCTB) is a benign, aggressive, and osteolytic bone tumor, which mainly occurs in young people and causes severe bone destruction. GCTB is a rare tumor that typically occurs in long bone and spine. Even though GCTB is deemed a benign tumor, there are approximately 18% to 50% of local recurrence and 2% to 3% of metastases, mainly to the lungs.^[1–3]^

Histologically, GCTB is composed of sheets of neoplastic ovoid mononuclear cells expressing receptor of NF-kappaB ligand (RANKL), mononuclear cells of myeloid linage, and osteoclast-like giant cells of randomly distributed population both with high RANKL expression.^[4]^ With the detection of the RANKL signal transduction pathway, its role in the regulation of bone growth and turnover has become increasingly prominent. As a fully human monoclonal antibody, Denosumab specifically suppresses osteolysis by binding to RANKL.^[5]^ Denosumab has been approved by food and drug administration and European Medical Agency for osteoporosis and prevention of bone-related events in bone metastasis of solid tumors. Sunitinib is a multi-target tyrosine kinase inhibitor, and its extensive activity will show great potential in anti-angiogenic and direct antitumor therapy, especially in the treatment of gastrointestinal stromal tumors (GIST) and renal cell carcinoma.^[6,7]^ However, non-GIST sarcomas are rarely involved. This rare case is about a patient with giant cell tumor of the spine with lung and bone metastases. Denosumab with Sunitinib treatment in such patient has not been reported as far as we know.

## 2 Case presentation

**Figure 1:**
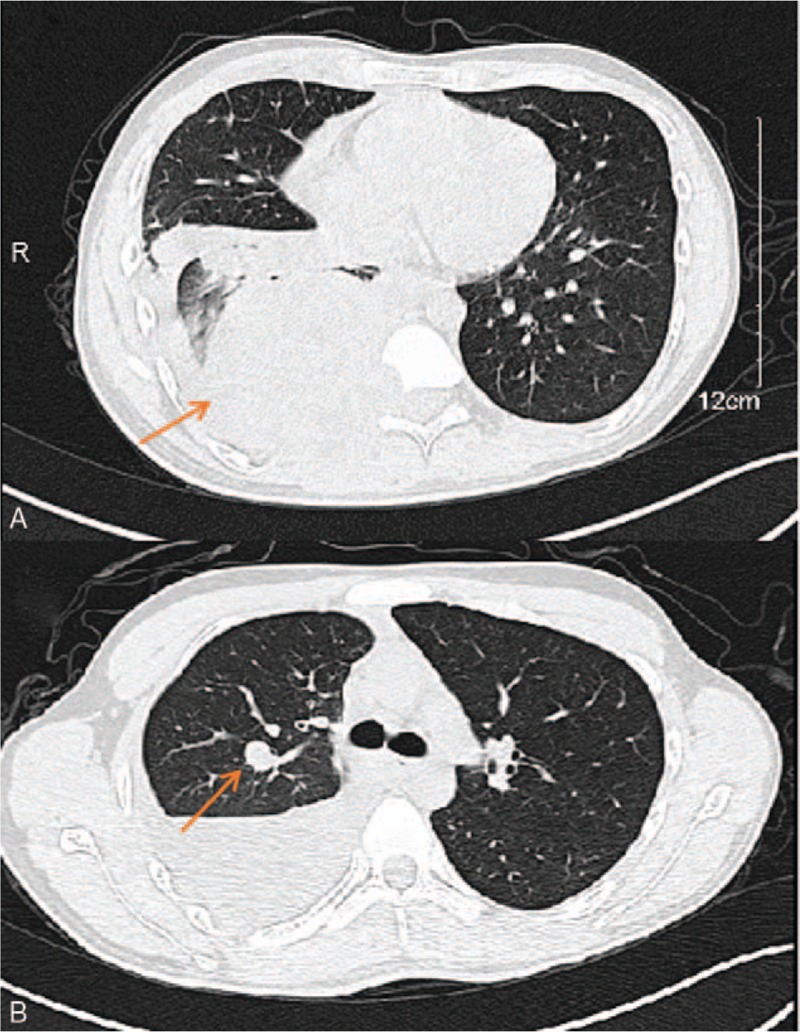
Axial CT study showing the soft tissue mass that involves the adjacent vertebrae (A) and metastasis in the posterior segment of the upper lobe of the right lung (B). CT = computed tomography.

**Figure 2:**
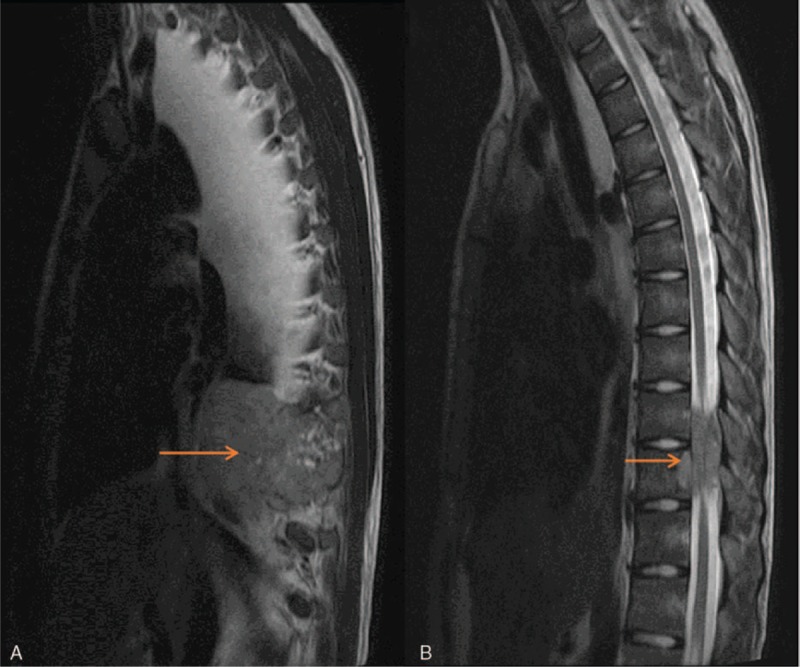
Preoperative sagittal MRI studies showing tumor completely violating the thecal sac and involving the adjacent vertebrae (A, B). MRI = magnetic resonance imaging.

**Figure 3:**
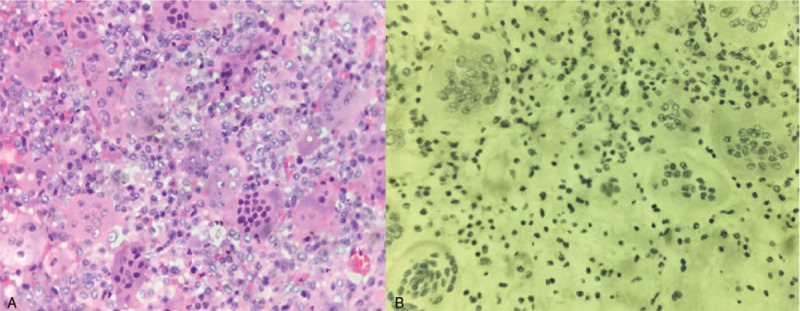
(A) Multinucleated osteoclast giant cells with large number of nuclei are evenly scattered among mononuclear tumor cells. (B) Mononuclear tumor cells display nuclear reactivity for P63.

A 16-year-old boy presented to our hospital in September 2014 for fever, chest tightness, shortness of breath, and back pain for 5 days. The patient had no neurological impairment. Computed tomography (CT) showed a soft tissue mass of 91 × 107 × 103 mm, involving the adjacent vertebrae and the ninth and tenth ribs on the right side of the T9–T10, as well as a node in the posterior segment of the upper lobe of the right lung (Fig. 1). Thoracic magnetic resonance inspection showed that the paravertebral heterogeneous tumor compressed the spinal cord (Fig. 2). Pathology of CT-guided pulmonary puncture indicated giant cell tumor of bone (right lung tissue) (Fig. 3). During hospitalization, the patient gradually developed movement limitation and hypoesthesia of both lower limbs and incontinence of stool and urine. The patient then underwent tumor resection and fixation-reconstruction via the posterolateral approach from T9 to T11 in another hospital. Postoperative pathology suggested giant cell tumor of bone. According to the tumor grade, site and metastases the patient was diagnosed as Enneking stage 3 giant cell tumor of spine. No adjuvant treatment was given after operation and the function and feeling of lower extremities were gradually improved after rehabilitation training. The lesions of lungs and ribs still existed.

Just 2 months later, the patient developed the same symptoms of paralysis of 2 lower limbs and incontinence and the recurrence of T8–T9 tumor accompanied by multiple pulmonary metastases. Then the second tumorectomy and fixation-reconstruction from T8 to T9 were performed and postoperative pathology still showed giant cell tumor of bone.

**Figure 4:**
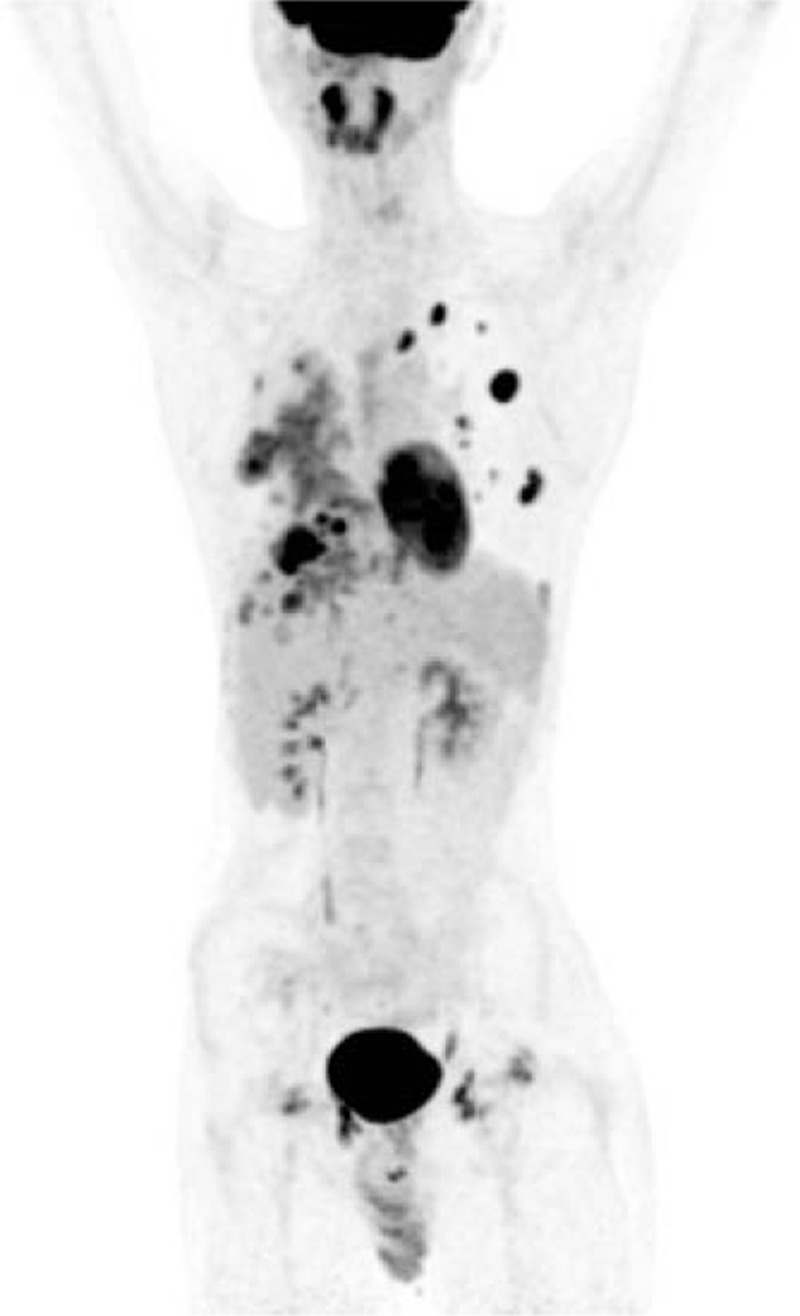
Positron emission tomography-computed tomography scanning showing that hypermetabolism in both lungs and the 9th and 10th ribs on the right.

After the operation, the patient began to receive 120 mg of subcutaneous Denosumab every 4 weeks with loading doses on the 8th and 15th day of the first cycle, and underwent chest image every 2 months to detect the lesions of lungs and ribs. After 4 months of treatment, his back pain did not improve significantly. The Positron emission tomography-computed tomography scanning indicated that multiple lesions in bilateral pulmonary increased and enlarged and radioactive uptake was also abnormally high (Fig. 4). So the curative effect of the patient was evaluated as progressive disease (response evaluation criteria in solid tumors version 1.1).

To reverse this trend, oncologists recommended gene testing in tumor tissues to determine whether there were gene overexpressions and mutations. Therefore, the high expression of the mRNA of platelet-derived growth factor receptor (PDGFR) β, vascular endothelial growth factor receptor (VEGFR)1, VEGFR2, VEGFR3, mammalian target of rapamycin was detected. According to the results, Sunitinib was added to the treatment strategy, which was administrated at 37.5 mg orally once daily. Within 5 months of the treatment with Denosumab and Sunitinib, his pain was dramatically improved and multiple pulmonary metastases shrunk.

Until now, after about 4 years of treatment, he could walk on crutches and growth and development were similar to his peers. There were no Denosumab-related adverse events or complications, and the adverse events associated with Sunitinib were less than grade 2 (common terminology criteria for adverse events version 4.0) including controllable nausea, erythema, and stomatitis. In the meanwhile, the patient took oral calcium and vitamin D daily, and the concentration of calcium and phosphorus were measured regularly and was in the normal range.

## 3 Discussion

GCTB with pulmonary and bone metastases at presentation make our patient unique. Surgery is still a main treatment for GCTB, with approximately 80% of patients receiving surgery. However, in multiple, metastatic and inoperable GCTB, surgery has limited use. Most patients probably suffer from disability after surgery, and the recurrence rate varies between 10% and 75%, depending on the size, location, and operation method.^[8]^ Though the literature on bisphosphonates, interferon-alfa, cytotoxic chemotherapy in the treatment of GCTB have been reported, the efficacy and side effects were not clear. Thomas et al^[9]^ was the first one to report the efficacy of Denosumab in GCTB, and then Chawla et al^[10]^ reported that the safety and efficacy of Denosumab, in which significant effects and predictable adverse events provided a reference for GCTB treatment. Our patient was treated with Denosumab as an adjuvant therapy after 2 surgeries, but the radiology showed progress in pulmonary metastases within 4 months of treatment, which left us confused and suspicious of the pathological results. Because in Thomas's study, we find that 2 patients dropped out of the treatment cohort on account of the progress caused by malignant GCTB.^[9]^ Therefore, in order to verify the pathological results, the second surgical specimen was sent to the department of pathology at the University of California, Los Angeles, and Professor Scott Nelson wrote: “the morphological characteristics of the tissue are most consistent with the typical giant cell tumor of bone.” Although malignant GCTB is rare, accounting for 1.8% to 7% of all GCTBs, it still has the potential to transform malignant especially after radiotherapy or several recurrences and surgeries.^[11]^ As specimens of lung metastases were not available, it is not possible to determine whether pathological changes occurred. In other words, the efficacy of Denosumab in the treatment of giant cell tumor of bone is uncertain because there is no clear definition of benign and malignant giant cell tumor of bone. Lau et al^[12]^ found that Denosumab caused only minimal inhibitory effects on the stromal cell lines of giant cell tumor and did not cause any apoptosis. Another possibility is that the patient had primary resistance to Denosumab, which resulted in metastatic progression. But until now there is a lack of clinical data about primary and secondary resistance of Denosumab. Although Denosumab did not control metastasis in this case, it may be effective in controlling local recurrence. So we cannot rule out Denosumab completely but it is not clear how long it will take for the drug to exhibit maximum effectiveness and how safe it will be for long-term use.

The addition of Sunitinib to the treatment strategy is the highlight of this case. Sunitinib, as a multitargeted tyrosine kinase inhibitor, targets VEGFR-1, 2, 3, PDGFR-α,β, KIT (CD117), fms-like tyrosine kinase 3, RET, and colony-stimulating factor-1. It is a representative of the anti-angiogenesis drugs and was approved for using in GIST and renal cell carcinoma treatment.^[13]^ To our knowledge, only 1 multicenter phase II trial observed Sunitinib in the treatment of non-GIST sarcoma. Though in the trial only 1 patient with advanced GCTB was enrolled, it was stable disease for at least 68 weeks.^[14]^ Fortunately, the pulmonary metastases of the patient in our case were also controlled with the Sunitinib. Therefore, Sunitinib may have great potential in the treatment of advanced giant cell tumor of bone based on the results of gene detection, which needs to be confirmed by further research.

In summary, we present an interest case of an adolescent GCTB with pulmonary and bone metastases which responded well to Denosumab and Sunitinib. However, there are still some questions about how long Denosumab and Sunitinib will last and how safe they will be. So this case will continue to be followed up and more clinical data and further studies are needed to explore new strategies for the treatment of metastatic GCTB.

## Acknowledgment

The authors thank all the group members for helpful discussions.

## Author contributions

**Data curation:** Sujing Jiang, Zhouqi Li.

**Resources:** Sujing Jiang, Zhouqi Li.

**Supervision:** Ying Dong.

**Writing – original draft:** Guannan Wang.

**Writing – review and editing:** Ying Dong.
